# The Association Between Onset of Staphylococcal Non-menstrual Toxic Shock Syndrome With Inducibility of Toxic Shock Syndrome Toxin-1 Production

**DOI:** 10.3389/fmicb.2022.765317

**Published:** 2022-03-14

**Authors:** Yusuke Taki, Shinya Watanabe, Yusuke Sato’o, Xin-Ee Tan, Hisaya K. Ono, Kotaro Kiga, Yoshifumi Aiba, Teppei Sasahara, Aa Haeruman Azam, Kanate Thitiananpakorn, Srivani Veeranarayanan, Feng-Yu Li, Yuancheng Zhang, Tomofumi Kawaguchi, Sarah Hossain, Maniruzzaman, Dong-Liang Hu, Longzhu Cui

**Affiliations:** ^1^Division of Bacteriology, Jichi Medical School, Tochigi, Japan; ^2^Department of Gastroenterological Surgery, Shizuoka General Hospital, Shizuoka, Japan; ^3^Department of Zoonoses, School of Veterinary Medicine, Kitasato University, Towada, Japan

**Keywords:** toxic shock syndrome, TSST-1, *Staphylococcus aureus*, *tst*, promoter mutation

## Abstract

Non-menstrual toxic shock syndrome (non-mTSS) is a life-threatening disease caused by *Staphylococcus aureus* strains producing superantigens, such as staphylococcal enterotoxins A, B, C, and toxic shock syndrome toxin-1 (TSST-1). However, little is known about why the TSS cases are rare, although *S. aureus* strains frequently carry a *tst* gene, which encodes TSST-1. To answer this question, the amount of TSST-1 produced by 541 clinical isolates was measured in both the presence and absence of serum supplementation to growth media. Then a set of *S. aureus* strains with similar genetic backgrounds isolated from patients presenting with non-mTSS and those with clinical manifestations other than non-mTSS was compared for their TSST-1 inducibility by human serum, and their whole-genome sequences were determined. Subsequently, the association of mutations identified in the *tst* promoter of non-mTSS strains with TSST-1 inducibility by human serum was evaluated by constructing promoter replacement mutants and green fluorescent protein (GFP) reporter recombinants. Results showed that 39 out of 541 clinical isolates (7.2%), including strains isolated from non-mTSS patients, had enhanced production of TSST-1 in the presence of serum. TSST-1 inducibility by human serum was more clearly seen in non-mTSS strains of clonal complex (CC)-5. Moreover, the whole-genome sequence analysis identified a set of sequence variations at a putative SarA-binding site of the *tst* promoter. This sequence variation was proven to be partially responsible for the induction of TSST-1 production by human serum. We conclude that the onset of staphylococcal toxic shock syndrome caused by TSST-1-producing CC-5 strains seem at least partially initiated by serum induction of TSST-1, which is regulated by the mutation of putative SarA-binding site at the *tst* promoter.

## Introduction

*Staphylococcus aureus* causes a wide variety of infectious diseases ranging from mild local infections, such as impetigo, to life-threatening diseases, including toxic shock syndrome (TSS) (Lowy, 1998). TSS is a systemic disease characterized by fever, hypotension, rash, desquamation, and multiple organ failures, such as gastrointestinal symptoms, renal dysfunction, liver failure, thrombocytopenia, and damage to the central nervous system (CDC, 2011). TSS is generally categorized into menstrual TSS (mTSS) and non-menstrual TSS (non-mTSS) depending on the cause of onset since there was a high incidence of TSS in menstruating women in the 1980s (Broome, 1989). According to the active surveillance of TSS reported by DeVries et al. (2011) the prevalence of TSS peaked at 13.7 per 100,000 in 1980, but has rapidly decreased to approximately 1.5–2.5 and 0.52 in 1986 and 2000–2003, respectively. Adams et al. (2016) described passive surveillance of TSS, and the prevalence of TSS was found to be at 0.03–0.05 between 2004 and 2014 with no remarkable change. Several factors were suggested to have caused the recent low prevalence of mTSS, such as the decrease in tampon absorbency, standardized absorbency labeling as required by the US Food and Drug Administration, concomitant education of TSS, and decreasing use of tampons (Hajjeh et al., 1999; DeVries et al., 2011). However, the rate of non-mTSS increased due to changes in delivery of surgical healthcare services, an increase in both outpatient procedures and the use of prosthetic devices, and increased hospitalizations caused by postoperative infections and infections from prosthetic devices (Simonsen et al., 1998; DeVries et al., 2011). Therefore, the rate of non-mTSS among all TSS cases increased from 9% in 1979–1980 (Hajjeh et al., 1999) to 46% in 2000–2006 (DeVries et al., 2011). Furthermore, even though the mortality rate of mTSS decreased gradually from 5.5% in 1979–1980 to 1.8% in 1987–1996 (Jenul and Horswill, 2019) and eventually to 0% in 2000–2006 (DeVries et al., 2011), that of non-mTSS remained high (8.5, 6, and 4%, respectively, for the three periods of time mentioned above). The difficulty in prevention and high mortality of non-mTSS render it one of the most problematic infectious diseases caused by *S. aureus*.

*S. aureus* strains that produce superantigens, such as toxic shock syndrome toxin-1 (TSST-1), staphylococcus enterotoxins A (SEA), staphylococcus enterotoxins B (SEB), and staphylococcus enterotoxins C (SEC) have been identified as the causative agents of TSS (Bergdoll et al., 1981). The detection rate of these superantigens were different between mTSS and non-mTSS. TSST-1, SEA, SEB, and SEC, in respective order, were detected in 89, 25, 0, and 13% of mTSS cases, whereas they were found in 50, 100, 60, and 25% of non-mTSS cases, respectively (DeVries et al., 2011). TSST-1 and enterotoxins act as a superantigen crosslinking between antigen-presenting cells and T-cell receptors; as many as 20% of the T cells are activated, leading to massive cytokine production (Low, 2013). The huge amount of cytokine causes systemic inflammations resulting in high fever, rash, desquamation, hypotension, and multiorgan failure. The most frequently detected superantigen, TSST-1, is coded by the *tst* gene on *Staphylococcus* pathogenicity island (SaPI) (Lindsay et al., 1998). *S. aureus* has an intricate regulatory network that controls the production of TSST-1, such as accessory gene regulator (Agr), staphylococcal accessory regulator (Sar), and small regulatory RNAs. The quorum-sensing *agr* system is a global virulence regulator of *S. aureus*, and it positively regulates the *tst* gene (Novick, 2003). Another global virulence factor SarA also regulates the *tst* gene expression by binding to the promoter region of *tst* (Chan and Foster, 1998; Andrey et al., 2010). However, SarA functions as both a positive and a negative regulator of *tst* depending on strain background (Andrey et al., 2015). Interestingly, *S. aureus* MN NJ strain derived from a non-mTSS patient showed upregulated *sarA* expression during the stationary phase of growth following supplementation of rabbit serum (Yarwood et al., 2002), indicating a potential interplay between TSST-1 production, *sarA* expression, and serum inducibility. RNAII constituting the *agr* operon indirectly affects *tst* regulations (Dunman et al., 2001). Furthermore, SigB regulates TSST-1 through an indirect mechanism involving two virulence regulators *agr* and SarA (Kusch et al., 2011; Andrey et al., 2015). The catabolite control protein CcpA, which senses glucose concentration in the environment, modulates *tst* expression by direct binding to a cognate catabolite-responsive element (*cre*) overlapping the *tst* transcription start site (Seidl et al., 2008; Andrey et al., 2010). Other environmental factors, such as oxygen, magnesium ion, pH, antibiotics, and vaginal microbiota, also affect the TSST-1 production (Chan and Foster, 1998; Ross and Onderdonk, 2000; Yarwood and Schlievert, 2000; MacPhee et al., 2013; Andrey et al., 2015).

As a well-recognized contributing factor of TSS, the *tst* gene is often found in clinical *S. aureus* sampled from TSS patients. *S. aureus* strains isolated from patients with mTSS frequently carry the *tst* gene (81.8–100%) (Bergdoll et al., 1981; Garbe et al., 1985; Lee et al., 1992), while the detection rate of *tst* gene is relatively lower in non-mTSS strains (62.5–68.3%) (Garbe et al., 1985; Lee et al., 1992). However, the *tst* gene seems not to be the sole determinant of TSS. *S. aureus* is part of the human skin or mucus microbiota, and 30–50% of healthy people are asymptomatically colonized by *S. aureus* (Lowy, 1998). Even though it has been reported that about 20% of the *S. aureus* strains are *tst* positive (Zhao et al., 2019), most of the *tst*-positive strains are opportunistic and do not cause TSS. Therefore, *tst*-positive *S. aureus* strains isolated from TSS patients may establish TSS through a yet-to-be-identified mechanism. Moreover, most of the previous studies were conducted using *S. aureus* strains that were not isolated from TSS patients. Hence, the regulatory mechanisms of *tst* in clinical strains isolated from TSS patients contributing to TSS development are still unclear.

To reveal the regulatory mechanisms of *tst* during the onset of TSS, we first analyzed TSST-1 production of clinical isolates of *S. aureus* by culturing the strains in a medium supplemented with horse serum to mimic an environment of onset of TSS. We found that horse serum induced TSST-1 production in some *tst*-positive *S. aureus* isolates, including strain Sak-1 (SCC*mec* type II, ST-5) (Cui et al., 1999) isolated from a fatal case of TSS. To investigate whether non-mTSS strains were TSST-1 inducible through supplantation of serum, human serum was used for induction assay on clinical strains in which clinical information is available. Similar to the case of Sak-1, two other clonal complex (CC)-5 strains isolated from postoperative TSS patients showed overproduction of TSST-1 by supplementation of human serum. Two of the three CC-5 strains isolated from non-mTSS patients have a mutation in the *tst* region responsible for the overproduction of TSST-1 following human serum induction. Our results indicate that the mutation on the *tst* promoter of CC-5 *S. aureus* may play a pivotal role in developing staphylococcal non-mTSS.

## Materials and Methods

### Bacterial Strains and Growth Conditions

A total of 541 clinical strains of *S. aureus*, including 256 methicillin-resistant *S. aureus* (MRSA) and 285 methicillin-susceptible *S. aureus* (MSSA), isolated from patients with various diseases between 1981 and 1996 from 10 medical institutions in Japan were included in the analysis of TSST-1 production. We have also analyzed seven *tst*-positive strains isolated from non-mTSS patients between 1999 and 2017 in Japan (Table 1) and seven *tst*-positive strains isolated between 2015 and 2018 in Japan from patients who did not develop TSS. The diagnosis of TSS was made clinically based on the CDC criteria (CDC, 1990; Supplementary Table 1). In addition, N315 (MRSA, ST-5) isolated from patients who did not develop TSS was included as a control strain (Kuroda et al., 2001).

**TABLE 1:** Characteristics of 15 *Staphylococcus aureus* strains used in this study.

	Clinical information		Genotype			Staphylococcal superantigen gene^a^											
			
Strain	Institution^b^	TSS^c^	CC^d^	ST^e^	SCC*mec* type	*sea*	*seb*	*sec*	*seg*	*sei*	*sell*	*selm*	*seln*	*selo*	*selp*	*selu*	*tst*
Sak-1	A	+	CC-5	ST-5	Type II	−	−	+	+	+	+	+	+	+	−	+	+
JMUB3007	B	+	CC-5	ST-5	Type II	−	−	+	+	+	+	+	+	+	−	+	+
JMUB3024	C	+	CC-5	ST-5	MSSA	−	−	+	+	+	+	+	+	+	+	+	+
N315	D	−	CC-5	ST-5	Type II	−	−	+	+	+	+	+	+	+	+	+	+
JMUB4687	E	−	CC-5	ST-2809	Type II	+	−	+	+	+	+	+	+	+	−	+	+
JMUB4716	E	−	CC-5	ST-2809	Type II	+	−	+	+	+	+	+	+	+	−	+	+
JMUB3011	F	+	CC-8	ST-8	Type IV	−	−	+	−	−	+	−	−	−	+	−	+
JMUB3035	G	+	CC-8	ST-8	MSSA	−	−	+	−	−	+	−	−	−	−	−	+
JMUB3036	H	+	CC-8	ST-8	MSSA	−	−	+	−	−	+	−	−	−	+	−	+
JMUB4633	E	−	CC-8	ST-8	Type IV	−	−	+	−	−	+	−	−	−	+	−	+
JMUB4688	E	−	CC-8	ST-8	Type IV	−	−	+	−	−	+	−	−	−	−	−	+
JMUB4700	E	−	CC-8	ST-8	Type IV	−	−	+	−	−	+	−	−	−	+	−	+
JMUB3038	I	+	CC-30	ST-30	MSSA	+	−	−	+	+	−	+	+	+	−	+	+
JMUB4692	E	−	CC-30	ST-30	MSSA	−	−	−	+	+	−	+	+	+	−	+	+
JMUB4641	E	−	CC-30	ST-30	MSSA	+	−	−	+	+	−	+	+	+	−	+	+

*^a^Representative superantigen genes identified on the genome sequence are listed.*

*^b^Medical institution where the S. aureus strains were obtained are anonymized.*

*^c^TSS denotes a bacterial strain that was isolated from a toxic shock syndrome patient.*

*^d^CC, Clonal complex.*

*^e^ST, Multi-locus sequene type (MLST). + indicates the strain which possessed the corresponding toxin. − indicates the strain which did not possess the toxin.*

*S. aureus* strains used in this study were cultivated in Todd Hewitt (TH) broth (Becton, Dickinson and Company; BD, NJ, United States), brain heart infusion (BHI) broth (BD), or BHI agar (BD) with aeration at 37°C, unless indicated otherwise. *Escherichia coli* DH5α and BL21 were aerobically grown in Luria–Bertani (LB) broth (BD) and tryptic soy agar (TSA, BD) at 37°C. In order to maintain the plasmids of *S. aureus* and *E. coli*, 10 μg/ml of chloramphenicol (Nacalai Tesque, Inc., Kyoto, Japan) was supplemented in culture medium. The bacterial culture was stocked at −80°C in a final concentration of 50% glycerol (Wako Pure Chemical Industries, Ltd., Tokyo, Japan) until further use.

### Detection of *tst* Gene by PCR

*S. aureus* isolates grown overnight in BHI broth were lysed by lysostaphin (Merck KGaA, Darmstadt, Germany) and achromopeptidase (Wako Pure Chemical Industries, Ltd.). PCR was performed by Quick Taq ^®^ HS DyeMix (Toyobo Co., Ltd., Osaka, Japan) using the cell lysate as a template. Primer set GTSSTR-1 and GTSSTR-2 (Supplementary Table 2) was used to amplify a 326-bp region of *tst*. The thermal cycling conditions included initial denaturation at 95°C for 3 min followed by 35 cycles of 98°C for 10 s, 50°C for 30 s, and 68°C for 1 min. The amplified products were electrophoresed on 1% agarose gel, stained with ethidium bromide, and visualized using AE-6933FXES Printgraph (Atto Co., Tokyo, Japan).

### Whole-Genome Sequencing

Phenol–chloroform-based extraction of genomic DNA from *S. aureus* and purification of DNA using a QIAamp DNA mini kit (Qiagen, Hilden, Germany) was described previously (Watanabe et al., 2018). Genomic sequencing and assembly were performed as previously described with slight modifications (Watanabe et al., 2019), and default parameters were used except otherwise noted. Briefly, sequencing was performed using a combination of Oxford Nanopore Technologies (ONT) platform [MinION Mk-1B devise (ONT, Oxford, United Kingdom) integrated with a FLO-MIN106 (R9.4.1) flow cell (ONT)] and Illumina MiSeq platform [Nextera XT library preparation kit (Illumina) and the MiSeq reagent kit version 3 (Illumina)]. Raw data obtained from the ONT platform were base called and demultiplexed by the MinKNOW software (ONT) and porechop (version 0.2.41^1^), respectively. After read-trimming by seqkit (v0.10.1) (Shen et al., 2016), the reads were *de novo* assembled into single contigs by flye (v2.8.1-b1676) (Kolmogorov et al., 2019) and racon (v1.4.12) (Vaser et al., 2017). The assembled sequences were polished with short-read sequencing data of MiSeq by pilon (v1.22) (Walker et al., 2014). The collected whole-genome sequences were finally annotated by prokka (v1.14.6) (Seemann, 2014). Assembled genome sequences of 998 *tst*-positive *S. aureus* strains were downloaded from the National Center for Biotechnology Information (NCBI).^2^ Phylogenetic analysis was performed by kSNP3.0 (Gardner et al., 2015).

### Quantification of Toxic Shock Syndrome Toxin-1

Frozen stock of *S. aureus* strain was revived in 4 ml of BHI broth for one night. To measure TSST-1 production without induction, 2 μl of the overnight culture was added into 1 ml of BHI broth. For serum induction assay, 2 μl of the overnight culture was added into 200 μl of BHI broth supplemented with 800 μl of horse serum (Thermo Fisher Scientific, MA, United States) or pooled human serum (Kohjin Bio Co., Ltd., Saitama, Japan). The culture was incubated with shaking at 37°C in 5% CO_2_ for 16 h.

TSST-1 production was determined by reversed passive latex agglutination kit (TST-RPLATM, Denka Seiken Co., Ltd., Tokyo, Japan) or sandwich enzyme-linked immunosorbent assay (ELISA). Latex agglutination assay has a detection limit of 1–2 ng/ml. In latex agglutination assay, 25 μl of twofold serially diluted culture supernatant was mixed with an equal volume of latex particles conjugated with antibodies against TSST-1 in V-bottomed microtiter plates. After 20 h of incubation at room temperature, the agglutination reaction was observed. An *S. aureus* strain was considered positive for TSST-1 production if aggregation was observed in wells with titers greater than 2. For ELISA assay, rabbit anti-TSST-1 antibody was used as a capture antibody, and chicken anti-TSST-1 immunoglobulin Y was used as a detection antibody. Using the avidin–biotin system, o-phenylenediamine’s absorbance was detected at a wavelength of 490 nm with VersaMax microplate reader (Molecular Devices, San Jose, CA, United States).

### Reporter Assay for Analysis of *tst* Promoter Activity

To assess the activity of *tst* promoter, the *tst* promoter regions of representative strains (N315, JMUB2909, JMUB3011, and JMUB3038) were amplified by PCR using the primer set pKAT-N315-*tst*-prom-F2 and *egfp*-N315-*tst*-prom-R (Supplementary Table 2). In addition, *egfp* and pKAT vector (Kato et al., 2017) were amplified with primer sets *egfp*-F/pKAT-*egfp*-R and pKAT-F/pKAT-R, respectively (Supplementary Table 2). The three PCR fragments were assembled by NEBuilder (New England Biolabs, MA, United States) to construct four plasmids: pKAT-9T-tst-promoter-gfp, pKAT-8T-tst-promoter-gfp, pKAT-7T-tst-promoter-gfp, and pKAT-6T-tst-promoter-gfp. These plasmids were transformed into *E. coli* DH5α. After extraction, the plasmids were electroporated into competent cells of N315 strain, as described previously (Sato’o et al., 2018). One microliter of the precultured N315 transformant was transferred into each 100 μl of BHI supplemented with 400 μl of human serum and 500 μl of BHI. A final concentration of 10 μg/ml of chloramphenicol was added into each medium to maintain plasmids. After 16 h of incubation, each culture was centrifuged at 10,000 rpm for 10 min, and the pellet was dissolved in 50 μl of PBS. The fluorescence was measured at an emission wavelength of 538 nm after excitation with a wavelength of 485 nm by microplate fluorometer Fluoroskan Ascent FL (Thermo Fisher Scientific). The fluorescence intensity was normalized by the turbidity of the bacterial culture.

### Construction of Recombinant *Staphylococcus aureus* Strains Carrying a Mutation in *tst* Promoter

The *tst* promoter regions of JMUB3007 and N315 strains were exchanged using a temperature-sensitive plasmid vector pIMAY (Monk et al., 2012). The *tst* promoter regions of both strains were amplified by PCR using primers pIMAY-*tst*-UP and pIMAY-*tst*-DN2 (Supplementary Table 2). On the other hand, pIMAY was amplified with primer set pIMAY-DW-F and pIMAY-DW-R (Supplementary Table 2). The DNA fragments were assembled by In-Fusion HD cloning kit (Takara Bio Inc., Shiga, Japan) and transformed, first, into *E. coli* DH5α and followed by *E. coli* BL21. *E. coli* B derivative BL21 strain was used as an intermediate to increase *S. aureus* transformation efficiency as our previous research has shown that the plasmids extracted from BL21 have an increased transformation efficiency of > 2 log10 folds (Sato’o et al., 2018). After extraction, the plasmids were electroporated into the respective *S. aureus* strains. The plasmid was then removed by culturing the transformants in a medium supplemented with anhydrotetracycline. The sequences of the replaced *tst* promoter regions were confirmed by Sanger-sequencer 3730xl DNA Analyzer (Thermo Fisher Scientific).

### Construction of *sarA*-Knockout Strain and Reporter Assay for Analyzing *tst* Promoter Activity

The *sarA* gene of the N315 strain was eliminated using temperature-sensitive plasmid pIMAY. About 1-kbp upstream and downstream flanking DNA fragments of *sarA* were amplified by PCR using primer sets pIMAY-sarAKO-DN1/sarAKO-fPCR-UP1 and pIMAY-sarAKO-UP1/sarAKO-fPCR-DN1 (Supplementary Table 2). A DNA fragment comprising a pIMAY vector backbone was amplified with primers pIMAY-DW-F and pIMAY-DW-R2 (Supplementary Table 2). The three DNA fragments were assembled by In-Fusion HD cloning kit and transformed into *E. coli* BL21. After extraction of knockout plasmid pIMAY sarAKO, the plasmid was electroporated into N315. The *sarA* gene of N315 was then eliminated by allelic exchange as described previously (Monk et al., 2012). The sequence of N315 Δ*sarA* was confirmed by Sanger-sequencer 3730xl DNA Analyzer. Finally, pKAT-9T-tst-promoter-gfp and pKAT-8T-tst-promoter-gfp were electroporated into N315 Δ*sarA* for use in reporter assay.

Reporter assay was performed in 50 μl of total reaction volume. Briefly, 0.2 μl of overnight culture was inoculated into 10 μl of BHI supplemented with 40 μl of human serum or 50 μl of BHI; both culture medium contained 10 μg/ml of chloramphenicol for plasmid maintenance. After 16 h of incubation, each culture was centrifuged at 15,000 rpm for 5 min, and the pellet was resuspended in 50 μl of PBS. The fluorescence was measured at an emission wavelength of 538 nm after excitation with a wavelength of 485 nm by a microplate fluorometer Fluoroskan Ascent FL. The fluorescence intensity was normalized against the turbidity of the bacterial culture.

### Statistical Analysis

Student’s *t*-test was used for the analysis of normally distributed continuous variables.

### Data Availability Statement

The genome sequence data were registered in the DNA Data Bank of Japan (DDBJ).^3^ The accession numbers of CC-5 *S. aureus* Sak-1, JMUB3007, JMUB3024, JMUB4687, and JMUB4716 are DRR303938, DRR307742, DRR307743, DRR307744, and DRR307745, respectively. All accession numbers for *S. aureus* used in this study are shown in Supplementary Table 3.

### Ethics Statement

The study protocols were approved by the institutional review board of Shizuoka General Hospital (SGHIRB #2016091). Consent to participate was not required, following the ethical guidelines for medical and health research involving human subjects by the Ministry of Health, Labor and Welfare, Japan, since this study analyzed bacteria, which were isolated as clinical specimens, and patients’ personal health information could not be accessed.^4^ Bacterial isolates were taken from hospitals as part of the standard patient care and used anonymously.

## Results

### Serum Induction of Toxic Shock Syndrome Toxin-1 Production

**FIGURE 1:**
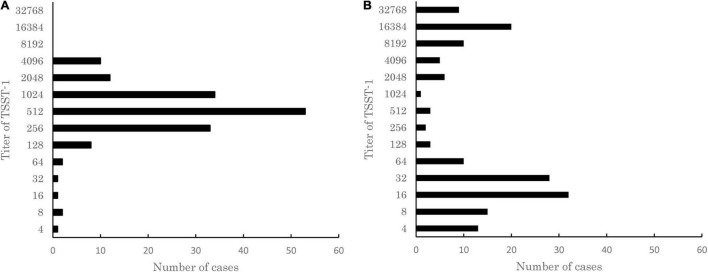
Distribution of titer of toxic shock syndrome toxin-1 (TSST-1) produced in 157 TSST-1-producing clinical isolates of *Staphylococcus aureus* cultured in **(A)** Todd Hewitt (TH) medium or **(B)** TH medium supplemented with horse serum. The titer of TSST-1 was determined by using the passive latex agglutination kit. The results of a single representative experiment carried out with three technical replicates are shown. Note that unimodal distribution of TSST-1 titers turned into bimodal distribution when the strains were cultured in the medium supplemented with horse serum.

*S. aureus* has a complex regulatory system controlling *tst*, which varies with the strain background (Novick, 2003; Andrey et al., 2015). In order to identify the specific bacterial populations, which can be triggered to produce TSST-1 during infection, we analyzed 541 clinical *S. aureus* strains isolated from patients with various diseases between 1981 and 1996 from 10 medical institutions in Japan. First, we assessed the ability of the 541 strains to produce TSST-1 under *in vitro* conditions by passive latex agglutination kit. A total of 157 out of the 541 strains (29%) produced TSST-1 after overnight culture in TH broth. The prevalence of TSST-1-producing strains was similar to previous data, for example, 22.7% of strains isolated in China were found to be able to produce TSST-1 (Zhao et al., 2019). The measured TSST-1 titer showed unimodal distribution from 4 to 4,096 with a median of 512 (Figure 1A). Next, we evaluated if the production of TSST-1 by TSST-1-producing strains was inducible by serum. Supplementation of horse serum into culture medium altered TSST-1 production, and the titers of TSST-1 produced by the 157 strains were distributed from 4 to 32,768 with bimodal peaks at 16 and 16,384 (Figure 1B). Thirty-nine strains (24.8%) produced TSST-1 at titers of equal or more than 8, 192, and 88 strains (56.1%) produced TSST-1 at titers less than 32. These results indicated that the TSST-1-producing strains could be classified into three groups: (1) strains showing increased production of TSST-1 following supplementation of horse serum, (2) strains producing a similar amount of TSST-1 with or without supplementation of horse serum, and (3) strains showing decreased production of TSST-1 following supplementation of horse serum. Interestingly, six MRSA strains isolated from fatal TSS cases, including Sak-1 strain (see below), belonged to the first group, suggesting that there might be an association between TSST-1 induction by serum and development of TSS.

### Phylogenetic Analysis of *Staphylococcus aureus* Strains Isolated From Non-menstrual Toxic Shock Syndrome Cases

*S. aureus* strains having the ability to produce a high level of TSST-1 in the presence of serum include those isolated from fatal TSS cases, such as Sak-1, which we reported in 1999 (Cui et al., 1999). The Sak-1 strain was isolated from a postoperative TSS patient who had passed away due to severe clinical symptoms. This strain was shown to produce 32 times more TSST-1 when induced by human blood. Therefore, we had suggested that the induction of TSST-1 by human blood might contribute to the development of non-mTSS (Cui et al., 1999). This hypothesis can be supported by the above TSST-1 induction experiments conducted with 157 TSST-1-producing strains. To further clarify this postulation, we carried out a follow-up study with seven *S. aureus* strains (inclusive of Sak-1) isolated from the non-mTSS patients between 1999 and 2017 in Japan (Supplementary Table 1). Genome analysis revealed that the seven non-mTSS strains belonged to three different sequence types (STs): ST-5 (three strains), ST-8 (three strains), and ST-30 (one strain). All TSS strains carried the TSST-1 gene (*tst*) as well as other enterotoxin genes (Table 1). This study also included a well-characterized *tst*-positive strain N315 (SCC*mec* type II, ST-5) and seven other *tst*-positive strains isolated between 2015 and 2018 in Japan from patients who did not develop TSS as controls. These *tst*-positive strains isolated from patients without TSS belonged to four different ST types: ST-5 (one strain), ST-2809 (two strains), ST-8 (three strains), and ST-30 (two strains).

**FIGURE 2:**
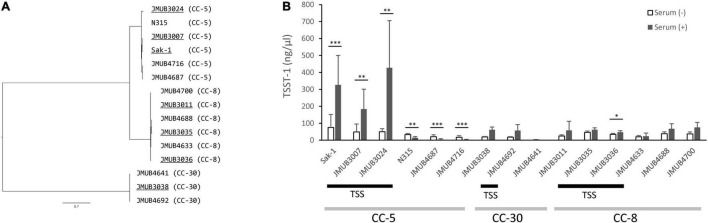
**(A)** Phylogenetic relationships among the *S. aureus* strains isolated from non-menstrual toxic shock syndrome (non-mTSS) patients (underlined) and patients who did not develop TSS (non-underlined). The consensus parsimony tree was constructed based on bead on SNPs determined by kSNP 3.1.2. Clonal complex (CC) types of the strains are presented in parentheses. **(B)** The amount of TSST-1 produced in *S. aureus* strains isolated from non-mTSS patients (TSS bar) or patients who did not develop non-mTSS when cultured in either brain heart infusion (BHI) broth (white bar) or BHI broth supplemented with human serum (black bar). The amount of TSST-1 produced was measured by enzyme-linked immunosorbent assay (ELISA) and is shown as means ± standard deviation. The level of significance, calculated by Student’s *t*-test, is denoted by asterisks (*, **, and *** indicate *p*-values of < 0.05, 0.01, and 0.001, respectively). Light gray bars denote CC types. The experiment was carried out 11 times in CC-5 strains and 4 times in CC-8 and CC-30 strains.

Phylogenetic analysis using whole-genome sequences showed that all the above strains could be roughly classified into three different CCs: CC-5, CC-8, and CC-30 (Figure 2A). Moreover, the phylogenetic analysis indicated that TSS strains and the strains isolated from patients without TSS were closely related to each other when they had the same ST. The ST-5 and ST-2809 strains belonged to CC-5, and they were classified into the same clade of the phylogenetic tree (Figure 2A).

### Induced Toxic Shock Syndrome Toxin-1 Production in Non-menstrual Toxic Shock Syndrome Strains

We assessed the ability of non-mTSS strains to produce increased TSST-1 after supplementation of human serum to growth media. The amount of TSST-1 produced by 15 representative strains in the presence and absence of human serum was measured and compared. As shown in Figure 2B, the induction of TSST-1 production by human serum varied among strains of different genotypes. The CC-5 strain Sak-1 had significantly higher TSST-1 production when human serum was added into the media. This result was in agreement with the previous study, which used whole blood as an inducing agent (Cui et al., 1999). Like Sak-1, other CC-5 strains isolated from TSS patients (JMUB3007 and JMUB3024) also produced a significantly increased amount of TSST-1 in the presence of human serum. In contrast, the CC-5 strains from patients who did not develop TSS had decreased TSST-1 production when human serum was added. We previously described that Mu50 (SCC*mec* type II, ST-5), isolated from patients who did not develop TSS, showed the same characteristics as our studied strains (Cui et al., 1999). On the other hand, there was no remarkable difference in TSST-1 production between the two different culture conditions for CC-8 and CC-30 strains regardless of whether the strains were isolated from TSS patients or not. These results indicated that the association between induced TSST-1 production and development of TSS would vary depending on the bacteria’s genetic background.

### Identification of a Mutation in the *tst* Promoter Region of Non-menstrual Toxic Shock Syndrome Strains

**FIGURE 3:**
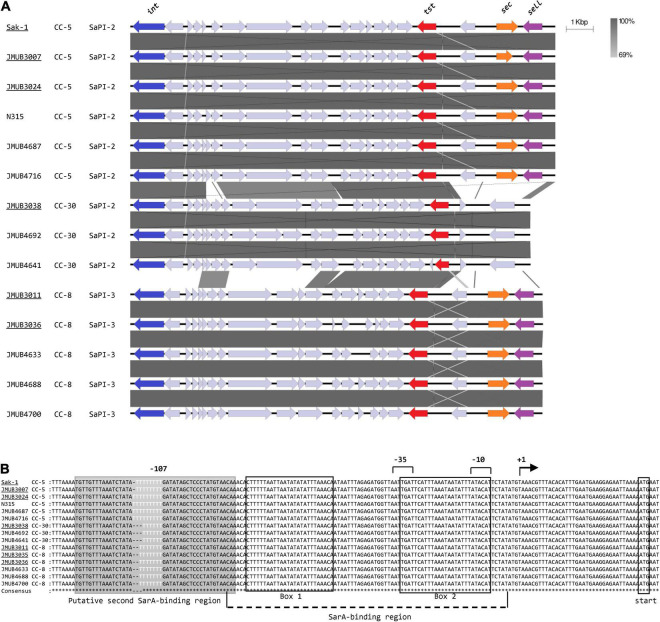
**(A)** Comparisons of *tst*-carrying *Staphylococcus* pathogenicity islands (SaPIs). The SaPI loci were extracted from the whole-genome sequence of each *S. aureus* strain included in Figures 2, 3 with the exception of JMUB3035 due to the lack of complete genome sequence and aligned for comparison. Strains isolated from non-mTSS patients are underlined. Note that the overall gene localizations, including the location of the *tst* gene, are well conserved among strains. **(B)** Alignment of the DNA sequences of the *tst* promoter regions. The bent arrow indicates the transcription start site of the *tst* transcript, as determined in the previous study (Andrey et al., 2010) (designated position +1). The −10 and −35 regions are indicated by numeral numbers, and the SarA-binding site and putative second SarA-binding site are shown in the box and shadowed box, respectively. The variable T repeat region is highlighted in white characters.

The *tst* gene is located on the *S. aureus* pathogenicity island (SaPI), which is highly diverse in structure and sequence (Lindsay et al., 1998). The whole-genome sequence analysis of the 15 strains revealed that the *tst* gene was located either on the SaPI2 in CC-5 and CC-30 strains or on the SaPI3 in CC-8 strains (Figure 3A). However, no remarkable difference was found in the SaPI structure between strains isolated from TSS patients and patients with clinical manifestations other than TSS (Figure 3A). The sequences of the coding region of *tst* were also found to be identical in all 15 strains. Nonetheless, sequence alignment of the *tst* promoter region showed that the number of thymidine (T) in the T repeat region at 107–115 bp upstream of the *tst* transcription start site (Andrey et al., 2010) varied from six to nine among the tested strains (Figure 3B). This region was supposed to be a putative second binding site of global regulator SarA. Among the six CC-5 strains, two of three CC-5-TSS strains [Sak-1 and JMUB3007 (SCC*mec* type II, ST-5)] carried eight consecutive T (8-T) repeats, while another CC5-TSS strain [JMUB3024 (MSSA, ST-5)] and the rest of the three strains from patients with clinical manifestations other than TSS carried nine consecutive T (9-T) repeats. The three CC-30 strains and six CC-8 strains carried 6- and 7-T repeats, respectively, regardless of whether the strains were isolated from TSS patients or not. The sequences of the *tst* promoter region, other than the T repeat region located 107–115 bp upstream of the *tst* transcription start site, were identical in all 15 strains. However, the induction of TSST-1 production by serum addition was different in each CC. Therefore, we hypothesized that the number of T’s in the region is involved in the regulation of TSST-1 and, thus, conducted an intensive study on the correlation of the number of T repeat with serum induction of TSST-1 production.

### Impact of T Repeat Mutation in *tst* Promoter Region on Serum Induction of Toxic Shock Syndrome Toxin-1 Production

**FIGURE 4:**
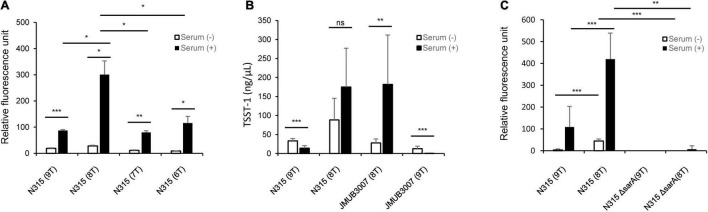
**(A)** Comparison of promoter activity among the eGFP overexpression mutants carrying *tst* promoter with different number of T repeats (–six to nine) in the putative second SarA-binding site. The relative fluorescence intensities are shown as means ± standard deviation. The experiment was carried out in triplicate. **(B)** Comparison of serum-induced TSST-1 production between *S. aureus* mutants carrying 8- and 9-T repeats in the putative second SarA-binding site of *tst* promoter. TSST-1 productions were measured by ELISA and shown as means ± standard deviation. The experiment was carried out five times. The level of significance, calculated by Student’s *t*-test, is denoted by asterisks. **(C)** Comparison of the activities of *tst* promoters with 8-T and 9-T repeats between wild-type and *sarA*-knockout N315 strain. The relative fluorescence intensities are shown as means ± standard deviation. The experiment was carried out in both technical duplicate and biological quintuplicate (*, **, and *** indicate *p*-values < 0.05, 0.01, and 0.001, respectively).

To confirm whether the number of T repeats in the *tst* promoter region affects serum induction of TSST-1 production, a reporter assay was performed using green fluorescent protein (GFP) expression vector pKAT (Kato et al., 2017). A set of *tst* promoter regions containing different number of T repeats (from six to nine) were individually cloned into the upstream of the *egfp* gene of pKAT to generate a series of eGFP expression plasmids, where the eGFP expression was regulated by the inserts. The constructed plasmids were then transformed into N315 (SCC*mec* type II, ST-5) strain, and the emitted fluorescence was measured with or without serum supplementation in the culture medium. As shown in Figure 4A, among all the transformants tested, N315 carrying a plasmid with 8-T repeats showed significantly increased eGFP expression compared with the others, a finding which was consistent with the results of serum induction of TSST-1 production observed in the clinical strains (Figure 2B). Since the CC-5 strains can be divided into two groups according to their TSST-1 inducibility, we also evaluated the influence of the number of T repeat on serum induction of TSST-1 using two CC-5 clinical strains, N315 (SCC*mec* type II, ST-5, carrying 9-T repeat promoter) and JMUB3007 (SCC*mec* type II, ST-5, carrying 8-T repeat promoter), by exchanging their *tst* promoters and subsequently culturing them with/without human serum. As expected, we found that serum induction significantly increased TSST-1 production when the 9-T repeat promoter was replaced with 8-T repeat promoter in N315, and vice versa, decreased when the 8-T repeat promoter was replaced with 9-T repeat promoter as seen in JMUB3007 (Figure 4B). These findings confirmed that the number of T repeats in the *tst* promoter region was at least one of the direct factors involved in regulating the serum induction of TSST-1 production.

### Influence of SarA on *tst* Promoter Activity

Since the T repeat region locates on the putative second SarA-binding region (Figure 3B), we investigated whether SarA positively or negatively regulates the *tst* expression of CC-5 strains using GFP reporter assay. A *sarA*-knockout N315 strain was constructed and transformed with plasmids carrying 9-T or 8-T repeat promoters upstream of *gfp* gene. Our results showed that *sarA* knockout strains have significantly decreased *tst* promoter activity even when they harbored 8-T repeat promoters, which was demonstrated earlier in this study to have a higher strength (Figure 4C). This result, hence, indicates that SarA is a positive regulator of *tst* gene in CC-5 strains.

## Discussion

Although the mortality rate of non-mTSS has been gradually decreasing over time, it is still a potentially fatal disease (Hajjeh et al., 1999; DeVries et al., 2011). The gene encoding TSST-1, *tst*, is widely distributed among *S. aureus* strains; however, the development of TSS is rare. The fact that a variety of conditions can contribute to its development, and the wide variation in disease manifestations from fulminant (Cui et al., 1999; Taki et al., 2016) to relatively mild course (Berger et al., 2019), make diagnosis difficult. We had previously reported a phenomenon of human serum-induced high TSST-1 production in a strain isolated from a severe TSS case (Cui et al., 1999). Here, we carried out a follow-up study to explore the relation between the development of TSS and induced TSST-1 production by serum and the possible mechanism underlying this induction. We observed that the level of serum-induced TSST-1 varied among the TSST-1-producing strains, and the high-level induction was seen only in *S. aureus* strains isolated from TSS patients. However, this observation was limited to strains with a particular genetic background, CC-5, for instance (Figure 2B). This serum-induced high-level production of TSST-1 was confirmed to be caused by a mutation in the promoter region of the TSST-1 toxin gene. We observed a considerable diversity in the number of T repeats in the *tst* promoter region and found that the difference in T repeat number affected the inducibility of TSST-1 by human serum. We also found that the SCC*mec* type II/ST-5 strains isolated from non-mTSS patients carried eight consecutive T repeats in the *tst* promoter. The eight T repeats provided *S. aureus* cells with the highest TSST-1-inducing ability. Our results suggest that the diversity of the *tst* promoter region has a clinical impact on TSS development.

**FIGURE 5:**
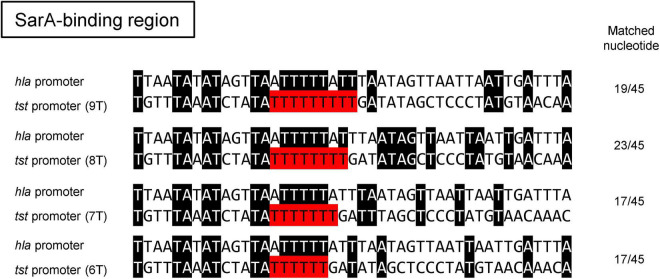
Sequence alignment of the SarA-binding site of *hla* promoter to those of the *tst* promoters with different number of T repeats in the putative second SarA-binding region. Note that the best-matched sequences (23 out of 45) between the promoters of *hla* and *tst* were found when the *tst* promoter was carrying eight consecutive repeats.

The T repeat variable region locates at 107–115 bp upstream of the *tst* transcription start site. The region was initially reported as the putative binding site of global regulator SarA due to its sequence similarity to other SarA-binding sites (Chien et al., 1999; Andrey et al., 2010). Two SarA-binding sites have been identified at 9–34 and 56–81 bp upstream of the transcription start site of *tst* (Andrey et al., 2010). The variation in the number of T repeats may affect SarA-binding activity because the *tst* promoter region with eight T repeats showed the highest similarity with the SarA-binding site of the *hla* gene encoding alpha hemolysin (Chien et al., 1999; Figure 5). This suggests that eight T repeats in the *tst* promoter might create an additional SarA-binding site, which provides the bacteria strains with serum-inducible TSST-1 production capability. Recently, Zhao et al. (2019) reported that some clinical isolates carried a different number of T repeats in the *tst* promoter region, and the *tst* expression differed among those strains. Our GFP reporter assay on *sarA*-knockout strain further confirmed the positive effect of SarA on *tst* promoter activity in CC-5 strains. Therefore, SarA might be the critical regulator of *tst*, which, in turn, modulates the development of TSS. However, additional study is needed to determine whether SarA directly binds the T-variable region and regulates *tst* gene.

**FIGURE 6:**
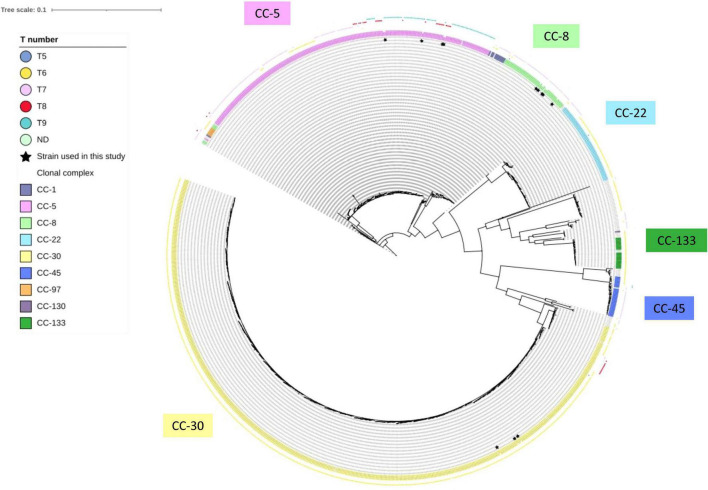
Parsimony-based phylogeny tree of 14 strains analyzed in this study and 998 *tst*-positive *S. aureus* deposited in NCBI. The color-shaded words denote the clonal complex of the bacterial strain. The color boxes of the outermost layer denote the number of consecutive T repeats in the putative second SarA-binding site of each strain. The strains with inner layer asterisk are the strains used in this study.

Nonetheless, strains with *tst* promoter harboring eight consecutive T repeats at the alternative SarA-binding site are relatively rare among the clinical *S. aureus* isolates. To confirm the prevalence of 8-T repeats, we extracted 998 assembled genome sequences containing the *tst* gene deposited in NCBI. The *tst*-promoter region was identified in 988 out of 998 strains (99.0%). Among them, the number of *S. aureus* strains carrying 9-T, 8-T, 7-T, 6-T, and 5-T repeats were 74 (7.5%), 36 (3.6%), 215 (21.8%), 660 (66.8%), and 3 (0.30%), respectively (Supplementary Table 4). We constructed a phylogenetic tree based on the genome sequences of *tst*-positive strains extracted from NCBI and strains analyzed in this study (Figure 6). The phylogenetic analysis revealed that 8-T repeats was confined to specific genetic backgrounds, including ST-5 (CC-5), ST-39, ST-37 (CC-30), and ST-88 (CC was not defined) (Figure 6). The low prevalence of *S. aureus* with eight consecutive T repeats may explain TSS cases’ rareness. However, *S. aureus* strains carrying different number of T repeats could be isolated from TSS patients (Figure 3B), and strains like JMUB3024 carrying 9-T repeats could respond significantly to serum induction (Figure 2B). It is also reported that host factors, such as antibodies against TSST-1, are associated with TSS development (Parsonnet et al., 2008). The regulations of onset of TSS caused by TSST-1-producing *S. aureus* are far from understood. Furthermore, this study focused only on CC-5, and MLST may affect SarA expression and other factors. Therefore, further study is needed to elucidate the comprehensive mechanisms of the development of TSS during *S. aureus* infection.

## Dedication

This article is dedicated to the memory of Keiichi Hiramatsu, Professor in the Department of Bacteriology, Juntendo University, who introduced the SW, YA, and LC to the field of Staphylococci and sadly passed away on June 5, 2020.

## Data Availability Statement

The datasets presented in this study can be found in online repositories. The names of the repository/repositories and accession number(s) can be found in the article/Supplementary Material.

## Author Contributions

YT, SW, and LC conceived and designed the study. YT and SW wrote the first draft of the manuscript. YT, SW, YS, X-ET, HKO, KK, YA, TS, AHA, KT, SV, F-YL, YZ, TK, SH, Maniruzzaman, and D-LH performed the experiments and collected the samples. YT, SW, and YS analyzed the data. X-ET and LC reviewed the manuscript. All authors read and approved the final manuscript.

## Conflict of Interest

The authors declare that the research was conducted in the absence of any commercial or financial relationships that could be construed as a potential conflict of interest.

## Publisher’s Note

All claims expressed in this article are solely those of the authors and do not necessarily represent those of their affiliated organizations, or those of the publisher, the editors and the reviewers. Any product that may be evaluated in this article, or claim that may be made by its manufacturer, is not guaranteed or endorsed by the publisher.

## Footnotes

1
https://github.com/rrwick/Porechop

2
https://www.ncbi.nlm.nih.gov

3
https://www.ddbj.nig.ac.jp/index.html

4
http://www.mhlw.go.jp/stf/seisakunitsuite/bunya/hokabunya/kenkyujigyou/i-kenkyu/index.html
