# Supplementary material for: The Association Between Onset of Staphylococcal Non-menstrual Toxic Shock Syndrome With Inducibility of Toxic Shock Syndrome Toxin-1 Production
Source: Front Microbiol. 2022 Mar 14;13:765317. doi: 10.3389/fmicb.2022.765317 (PMC8964310; doi:10.3389/fmicb.2022.765317)
Supplement: Supplementary file 3 [file Data_Sheet_1.docx]

**Supplementary Data**

**Table S2: Primer List**

| Name | Sequence |
| --- | --- |
| GTSSTR-1 | ACCCCTGTTCCCTTATCATC |
| GTSSTR-2 | TTTTCAGTATTTGTAACGCC |
| pKAT-N315-tst-prom-F2 | TTTATCCTTTACCTTGTCTccactagaaagtgactttaaagaatataact |
| egfp-N315-tst-prom-R | CTTCTCCTTTGCTAGCCATttttaattctccttcattcaaatgtgtaaacg |
| egfp-F | ATGGCTAGCAAAGGAGAAGAAC |
| pKAT-egfp-R | GGCACCGCCAGTGCCAAGCTCAATCGATGTTGTACAGTTCATCC |
| pKAT-F | GCTTGGCACTGGCGGTGCCTC |
| pKAT-R | AGACAAGGTAAAGGATAAAACAGCAC |
| pIMAY-tst-UP | TAGAGCGGCCGCCACCGataccatagaaggaacagctaatttag |
| pIMAY-tst-DN | AATACGACTCACTATAGGGCGttggctttttttagttctttttgtgtttaagtc |
| pIMAY-DW-F | CGCCCTATAGTGAGTCGTATT |
| pIMAY-DW-R | CGGTGGCGGCCGCTCTA |
| pIMAY-DW-R2 | gccgctctagaactagtggatc |
| pIMAY-sarAKO-DN1 | gatccactagttctagagcggccaaattgaaggtaaaggggatcc |
| sarAKO-fPCR-UP1 | ttttgtttagcgcaatttggtg |
| pIMAY-sarAKO-UP1 | AATACGACTCACTATAGGGCGgaaaagcgttgatttgggtagtatac |
| sarAKO-fPCR-DN1 | CACCAAATTGCGCTAAACAAAAgtttaaaacctccctatttgatgcatc |

**Table S3 The DDBJ accession numbers of all strains used in this study**

| Strain | Accession number (Miseq) | Accesion number (MinION) |
| --- | --- | --- |
| Sak-1 | DRR303938 | DRR337706 |
| JMUB3007 | DRR307742 | DRR337707 |
| JMUB3024 | DRR307743 | DRR337708 |
| JMUB4687 | DRR307744 | DRR337709 |
| JMUB4716 | DRR307745 | DRR337710 |
| JMUB3011 | DRR307746 | DRR337711 |
| JMUB3035 | DRR307747 |  |
| JMUB3036 | DRR307748 | DRR337712 |
| JMUB4633 | DRR307749 | DRR337713 |
| JMUB4688 | DRR307750 | DRR337714 |
| JMUB4700 | DRR307751 | DRR337715 |
| JMUB3038 | DRR307752 | DRR337716 |
| JMUB4692 | DRR307753 | DRR337717 |
| JMUB4641 | DRR307754 | DRR337718 |
